# Newborn Screening for Spinal Muscular Atrophy: Variations in Practice and Early Management of Infants with Spinal Muscular Atrophy in the United States

**DOI:** 10.3390/ijns10030058

**Published:** 2024-08-16

**Authors:** Craig M. Zaidman, Cameron D. Crockett, Ethan Wedge, Grace Tabatabai, Natalie Goedeker

**Affiliations:** 1 Department of Neurology, Washington University in St Louis School of Medicine, St. Louis, MO 63110, USA; ewedge@wustl.edu (E.W.); tabatabai.grace@wustl.edu (G.T.); ngoedeker@wustl.edu (N.G.); 2 Department of Neurology, University of Wisconsin School of Medicine and Public Health, Madison, WI 53705, USA; cdcrockett@wisc.edu

**Keywords:** spinal muscular atrophy, newborn screening, treatment practices, birth prevalence, gene therapy

## Abstract

In the United States (U.S.), newborn screening (NBS) for spinal muscular atrophy (SMA) is implemented by individual states. There is likely variation in the practice patterns of state NBS programs and among the providers caring for newborns with SMA. This is a prospective, descriptive, observational study that seeks to quantify and describe practice patterns and heterogeneities in state NBS programs and provider practices in the U.S. We surveyed U.S. state NBS programs and care providers of newborns with SMA. Thirty states and 41 practitioners responded. NBS program practices vary by state. Most (74%) state programs provide results to both primary care and specialist providers and also defer confirmatory SMA testing to those providers. Two states had relatively high rates of false-positive or inclusive results. The total birth prevalence of SMA was 1:13,862. Most providers were in tertiary care centers (90%) and were child neurologists (81%) and/or had fellowship training in Neuromuscular Medicine or Electromyography (76%). All providers see new referrals in less than a week, but many do not initiate treatment until >3 weeks of age (39%), with most commonly reported delays related to insurance processes. Most (81%) prefer onasemnogene abeparvovec-xioi (OA) as the treatment of choice, mainly due to perceived efficacy and the route/frequency of administration. NBS practice patterns in the U.S. vary by state but overall yielded the predicted birth prevalence of positive results. Providers evaluate these newborns urgently, but many do not initiate therapy until after 3 weeks of age. Treatment delays are mainly related to insurance processes.

## 1. Introduction

Spinal muscular atrophy (SMA) is a hereditary neuromuscular disease resulting from a recessively inherited mutation in the SMN1 gene on chromosome 5q. SMA is characterized by progressive weakness from deterioration of the anterior horn cells in the spinal cord and brain stem nuclei. Disease onset and severity ranges from before birth to adulthood, and generally correlates with the copy number of the SMN2 gene, a homologue to the SMN1 gene that produces a truncated protein. Traditional classifications categorized individuals with SMA as type 0–4 (more to less affected) based on age at symptom onset and the greatest motor function achieved. Landmark therapeutic achievements have altered the disease course of SMA. Three SMN protein-directed therapeutics are currently U.S. Food and Drug Administration (FDA)-approved for treatment of SMA based on improved outcomes in children with SMA [1,2,3,4]. Nusinersen (Spinraza^®^), an antisense oligonucleotide approved in December 2016, and risdiplam (Evrysdi^®^), a small molecule approved in August 2020 and expanded for use in newborns in May 2022, are both splice-site modifiers of SMN2 that lead to increased SMN protein production. Onasemnogene abeparvovec-xioi (OA) (Zolgensma^®^) is an AAV9 vector-based systemic, intravenous, SMN1 gene transfer therapy approved in May 2019 for use in children < 2 years of age.

Both preclinical and clinical data suggest that early treatment initiation in children with SMA is critical to achieving optimal outcomes. Preclinical data demonstrate early and severe denervation in the first months of life in children with SMA type 1 [5]. Symptomatic infants treated earlier with nusinersen or OA show improved functional outcomes compared to those treated later but still do not normalize motor development [3,6]. Initiating treatment in presymptomatic newborns with SMA leads to further improved outcomes. Newborns with SMA treated before symptoms develop are more likely to achieve head control, independent sitting, standing, and walking, and many children meet these gross motor milestones at developmentally appropriate ages [7,8,9]. These observations led to the inclusion of SMA in the federal Recommended Uniform Screening Panel in 2018. The current guidelines for the management of infants with SMA identified by newborn screening (NBS) indicate “immediate” treatment for NBS-identified infants with SMA and 2–4 copies of SMN2 [10].

In the U.S., NBS is implemented by individual states [11]. NBS for SMA in the U.S. was first implemented in 2018 and has recently expanded to include all 50 U.S. states and Washington, D.C. Each state determines the implementation plan, including the type of screening performed, the method for communicating results, and the method of short- and long-term follow-up of infants with positive NBS. Although the Centers for Disease Control and Prevention provide quality assurance directives to state programs for NBS, each state determines how to implement the program beyond the laboratory testing [11,12]. Nationwide SMA birth prevalence data stemming from state NBS programs are unknown.

The clinical backgrounds and practice patterns of providers caring for newborns with SMA are also mostly uncharacterized [13]. Variations in the experience and practice patterns of clinicians receiving referrals for newborns with SMA could impact outcomes, particularly given the urgency of treatment initiation in this patient population and the complexity of navigating the medical management and monitoring of a newborn with SMA.

The goal of this study is to quantify and describe heterogeneities in state NBS programs and provider practices for newborns with SMA across the United States. This information could help further inform guidelines on the practical implementation and management of NBS for SMA.

## 2. Methods

This is a prospective, descriptive, survey-based observational study. The Washington University Institutional Review Board has approved this study protocol and associated surveys with a waiver of informed consent (IRB ID #: 202101201, approval date 7 February 2021).

### 2.1. Survey of State NBS Programs

We developed a questionnaire to evaluate approaches to NBS for SMA. The 17-item questionnaire assessed various aspects of the NBS process, including testing methodology, screening results, follow-up protocols, and long-term outcomes. The full questionnaire is included in the Supplementary Materials.

We collected data from states that were either in the pilot phase for SMA NBS or had adopted mandatory statewide NBS for SMA prior to 31 January 2022. Offices were initially contacted by telephone, with questionnaires administered at the time of initial contact if possible. The offices were also allowed the opportunity to respond by email if preferred. Some offices were contacted for clarification on prior answers into the first quarter of 2022. Only those states which had entered an active phase of screening (i.e., were no longer in the pilot phase) at time of data collection were included in the data analysis. Not all respondents answered every question. Missing data points were excluded from the analysis. We reported the total number of respondents to each individual question and used this as the denominator to calculate percentages for responses to that question. We calculated birth prevalence per state as the number of NBS-positive SMA infants divided by the total number of infants screened in that state. We also calculated the total birth prevalence in our overall sample by dividing the total number of NBS-positive infants by the total number of infants screened using data aggregated only from states providing both values. Eight states did not provide sufficient data for birth prevalence calculation.

### 2.2. Survey of Providers Caring for Newborns with SMA

We distributed electronic surveys to practitioners who provide care to newborns with SMA. We obtained a list of centers from CureSMA (complete as of May 2022) who reported offering care for newborns with SMA and sent each site an electronic survey regarding their training and practice patterns. We solicited one survey from each care center. Each survey was directed to the provider who was primarily responsible for the care of newborns with SMA at each site or who could answer on behalf of the practice. We distributed the surveys in the second quarter of 2023 to 69 sites across the U.S. The surveys collected data regarding provider specialty and/or training, type of care center/practice, participation in clinical trials in SMA therapeutic development, treatment history and preferences, and patient treatment timelines. Several questions differentiated between care for newborns (defined as age < 2 months) and care for infants (defined as age < 1 year). Completion of all questions in the survey was required for submission. The full survey and results are included in the Supplemental Materials. For ranked-choice responses, we ranked each response according to its average ordinal score.

## 3. Results

### 3.1. Survey of State Newborn Screening Programs

#### 3.1.1. Participating States

**Figure 1:**
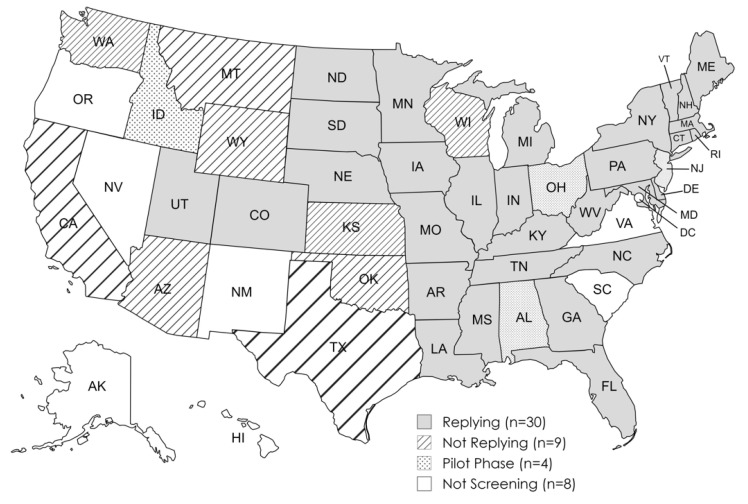
SMA newborn screen status of U.S. states and the District of Columbia as of 31 January 2022.

We surveyed state NBS programs in a total of 43 U.S. states. Thirty states responded to the questionnaire and had adopted mandatory statewide NBS for SMA prior to 31 January 2022. Data from these states were included in our analysis. Four states were in the pilot phase at time of data collection and were excluded from the data analysis. Nine states did not respond. Seven states and Washington, D.C. had not initiated screening for SMA at the time of data collection and were not contacted for this study. The states participating in our study are indicated in Figure 1.

#### 3.1.2. NBS Results

The median duration of SMA screening at the time of data collection was 19.5 months, ranging between 1 and 43 months. The total birth prevalence calculated across the 22 states who reported both the total number of infants screened and the number of positive screens was 1 in 13,862 (0.007%). The median state birth prevalence of SMA-positive infants identified by NBS was 1:20,000 (0.005%; range: 0.00–0.048%; 22 states reporting). Total and state SMA newborn screen results are further summarized in Table 1.

False positives were overall infrequent, with 21 states reporting no occurrences. Most (391/393) false positives occurred in two states: Georgia (364, 92.6% of false positives), where reporting did not differentiate inconclusive screens from true false-positive screens, and Arkansas (27, 6.9% of false positives). There were no reported false-negative screens.

#### 3.1.3. Testing Methodology and Approach to Follow-Up

State testing methodology and follow-up is summarized in Table 2. Most states do not perform confirmatory SMN1 genetic testing or SMN2 copy number testing. Most states performed screening for SMA in their own state-run laboratory (15/27, 56%), though regional labs (7, 25.9%) and commercial labs (5, 19%) were also used. One state (New York) reported the use of digital droplet PCR (ddPCR); others perform quantitative real-time PCR (qRT PCR).

Following a positive screen for SMA, most states contacted both the primary care provider and a specialist. Only a few contacted solely the primary care provider (three, 11%) or specialist (four, 15%). Most state screening programs performed some type of short-term follow-up of SMA-positive infants, such as ensuring the infant was referred to an SMA treatment center, following confirmatory testing results, or tracking treatment decisions (Table 2). Few states reported plans for long-term follow-up and data collection. Most states provide data to the Newsteps database (24/26, 92%), while fewer reported to the Newborn Screening Translational Research Network (6, 23%) or other entities (6, 23%)—most commonly the Association of Public Health Libraries (3, 12%).

### 3.2. Survey of Providers Caring for Newborns with SMA

#### 3.2.1. Respondent Characteristics

Forty-one of sixty-nine (59%) sites surveyed responded, representing 25 U.S. states, with all respondents completing every question of the survey. Most respondents (35, 85%) were the main care providers for newborns with SMA at their practice, while the remainder were providers who were able to answer on behalf of the practice. The survey results are summarized in Table 3.

Respondents were primarily based in tertiary care academic practices and provided care for mostly pediatric patients, including newborns with suspected neuromuscular diseases. Most respondents were child neurologists and had fellowship training in Neuromuscular Medicine or Electromyography. The training background of other respondents included pediatric pulmonology (one) and physical medicine and rehabilitation (one), and one respondent did not specify the training background of care providers (a care center director replying on behalf of the practice). All but one respondent had treated an infant with either nusinersen (40, 98%), OA (39, 95%), or risdiplam (34, 83%).

#### 3.2.2. Respondent Practice Patterns

Newborns identified with SMA on NBS were uncommon referrals. Most providers (28, 68%) reported evaluating 10 or fewer newborns with SMA in the last 24 months. Three (7%) had not received any newborn SMA referrals at the time of the survey. The providers report quickly evaluating newborns who screen positive for SMA, with most providers seeing these patients within 72 h of referral on average. All providers either perform confirmatory SMN1/2 gene testing in infants with positive NBS (39, 95%) or reported that confirmatory testing is already performed by the referring state NBS program. Most performed repeat/confirmatory genetic testing at the time of the initial clinic visit (34, 83%).

Most respondents reported treating newborns with SMA between 2 and 4 weeks of age on average (27, 66%, Table 3). None reported initiating treatment in ≤1 week on average. Most ranked insurance approval as the most time-consuming step in initiating treatment. Factors for the choice of starting versus deferring treatment, ranked from most to least important, were the presence of symptoms of SMA, SMN2 copy number, the efficacy of treatments, the side-effect profile, the insurance authorization process, and cost.

Respondents overwhelmingly reported OA monotherapy as their first-line recommendation for treatment (33, 81%, Table 3). The factors for determining the choice of treatment, ranked from most to least important, were treatment efficacy, route/frequency of administration, SMN2 copy number, the presence of symptoms of SMA, the side-effect profile, the mechanism of action, insurance authorization, and cost.

## 4. Discussion

NBS for SMA was initiated in the U.S. in 2018 and has recently expanded to include all 50 states and Washington, D.C. This study describes testing practices within state NBS programs as well as common practice patterns of providers caring for newborns with SMA within the United States [11].

We identified variations in the approach to NBS between states that could potentially impact either referral patterns or the timing of treatment initiation. First, some state NBS programs perform SMA confirmatory testing at the time of the initial screen, whereas others defer this practice to the treating provider, potentially delaying diagnostic confirmation. Second, while most NBS programs directly contact both the primary care provider and an identified SMA treatment specialist, some contact only the primary care provider or the SMA specialist alone. The potential lack of coordination between primary and specialty care providers at the time of result release could prolong the time to treatment. Third, although most states report no false-positive results, two states had relatively high numbers of inconclusive or false-positive results. Differences in false-positive/inconclusive rates likely result from individual lab variance in the cutoff values of the quantitative PCR cycle times. The rates of false-positive test results might impact provider decisions regarding initiating therapy pending confirmatory testing, which could delay access to treatment.

Our study yielded a total birth prevalence of SMA identified via NBS of 1:13,862. This is only slightly lower than the expected birth prevalence of 1:11,000 based on prior estimates [14,15,16]. Previous U.S. studies of NBS results of SMA were limited to individual states and yielded birth prevalence even lower than in our study [17,18,19]. We also found a lower-than-expected median state birth prevalence (1:20,000) compared to the total birth prevalence across our sample. It is likely that the lower-than-expected birth prevalence is related to the limited sample size rather than due to variation in testing performance between states. All NBS programs included in our study utilized either quantitative real-time PCR or digital droplet PCR. This technology is expected to identify the most common cause of SMA (homozygous deletion of the SMN1 gene) but will not detect the estimated 5% of children with SMA due to single-nucleotide variants [20]. No state in our sample reported a known false negative. False negative results may increase as screened infants age and affected children become symptomatic. This study is not able to assess the impact of prenatal testing on birth prevalence.

As SMA is one of the first pediatric neuromuscular disorders to be included in NBS in the U.S., it is likely that providers caring for children with neuromuscular disorders had to adopt new practice patterns to account for the specific requirements of this newly identified population. Referrals for newborns with suspected SMA remain a relatively rare occurrence, with most providers reporting 10 or fewer referrals within the last 24 months. Having relatively infrequent opportunities to care for this population may lead to lower levels of comfort in management. It is unclear from our current study how the infrequent nature of this referral might impact the approach to care. Despite the relatively infrequent nature of these referrals, providers do recognize the urgency of the initial evaluation for newborns with NBS-identified SMA given that all respondents reported seeing these patients within one week (most within 72 h). Despite this, many providers cannot initiate treatment until three weeks of life or later (39%), and no provider in our study reported the ability to initiate therapy within one week of life on average. When considering factors that led to delays in treatment initiation, most (83%) providers identified the insurance approval process as the most time-consuming step in managing these patients. These findings of perceived barriers to care are similar to a larger survey of SMA providers [13]. Because infants with SMA can develop symptoms in the first weeks of life, delays in treatment initiation could potentially impact outcomes [21].

Most (81%) providers identified OA as their treatment of choice, with perceived efficacy, route of administration, and frequency of administration ranked highest in factors for selecting treatment. This treatment preference for OA in patients identified via NBS is similar to real-world data practice patterns [22]. Despite providers’ perception of improved efficacy, there are no studies directly comparing the three SMN protein-directed therapies currently approved in the U.S. for treatment of SMA [23,24,25]. Two respondents selected a dual therapy regimen (risdiplam with OA) as a first-line recommendation for treatment. Few studies have described combination therapies for SMA [22,26,27,28]. Available evidence for adding risidiplam or nusinersen following treatment with OA suggests that this is well tolerated [29,30]. Cost was the lowest-ranking factor contributing to treatment decisions reported by providers.

Limitations of this study are common to observational survey-based studies. Our study may suffer from both selection and ascertainment bias. Our survey of state NBS programs captured states who were earlier adopters of screening for SMA and may not encompass more recent practices. Respondents to our provider survey were heavily weighted toward tertiary care practices, with most being child neurologists. It is possible that additional heterogeneity exists and was not captured by our survey. Evolving practice patterns (including potential lab method adjustments to reduce the number of infants with inconclusive results) would not be captured by our study. We delayed the survey of practice providers to capture practice patterns following the FDA approval of risdiplam in 2022 for use in patients with SMA less than 2 months of age. Although providers were generally experienced with all three SMN-directed therapies, given the rarity of newborn referrals for SMA and the recency of the expanded label for risdiplam, it is possible that providers in our survey had less experience using this medication.

The variability in the approach to NBS for SMA between states as well as in the clinical approach of providers caring for these patients highlights areas of potential inequality regarding the evaluation and management of this population. This study identified variations in state NBS programs’ performance, communication pathways, and providers’ triage and treatment initiation practices that could be standardized for consistency, potentially improving the overall care of NBS-identified patients with SMA. National consensus guidelines and educational programs specific for infants with SMA could help reduce these variations in practice patterns. Future efforts focusing on uniform standards for state NBS program accuracy and efficiency of communications and reducing barriers to timely treatment initiation are most likely to improve the equity of care of infants with SMA. Challenges remain regarding determining optimal therapeutic regimens and dispersing experiential knowledge beyond the tertiary care center. Patient registries and retrospective studies comparing outcomes in this relatively rare population are promising avenues to determine optimal treatment strategies.

## Figures and Tables

**Table 1:** U.S. SMA newborn screen results.

	Infants Screened (*N* = 22, Number of States Responding)	Positive Screens (*N* = 27)	False-Positive Screens (*N* = 25)	False-Negative Screens (*N* = 25)
Sample Total *	2,536,709	228	393	0
State Median (Range)	78,022 (2000–363,131)	7 (0–30)	0 (0–364)	0 (0–0)

* Note variation in number of respondents for each column.

**Table 2:** State SMA NBS program methodology and follow-up.

		# of States/# Responding (%)
**Testing Methodology**		
	Quantitative real-time PCR	23/24 (96%)
	Perform SMN2 copy number testing	12/28 (43%)
	Perform confirmatory testing of positive result	4/28 (14%)
**Positive NBS Screen Tracking/Follow-Up**		
	Communicate directly to both the primary care and SMA care provider	20/27 (74%)
	Track short-term course (referral to treatment center and confirmatory testing results)	24/27 (89%)
	Track treatment choice	21/25 (84%)
	Track longer term outcomes/longitudinal data collection	5/25 (20%)
	Participation in a NBS registry	26/26 (100%)

**Table 3:** Newborn SMA care provider survey results.

Total Respondents		*N* = 41
**Practice setting**		
	Tertiary care center—academic	37 (90%)
	Mostly pediatric	37 (90%)
	Provide care for newborns with neuromuscular disease	40 (98%)
	Has pediatric hospital admitting privileges	41 (100%)
	Participated in pediatric SMA clinical trials	19 (46%)
**Training background**		
	Child Neurology	33 (81%)
	Adult Neurology	5 (12%)
	Neuromuscular/EMG	31 (76%)
**Infant SMA therapeutic experience**		
	Nusinersen	40 (98%)
	Onasemnogene abeparvovec	39 (95%)
	Risdiplam	34 (83%)
	None	1 (2%)
**Time from referral to evaluation**		
	<72 h	28 (68%)
	Within one week	10 (24%)
	No referrals received	3 (7%)
**Average infant age at treatment**		
	<1 week	0 (0%)
	1–2 weeks	6 (14%)
	2–3 weeks	16 (39%)
	3–4 weeks	11 (27%)
	>5 weeks	5 (12%)
	None treated	3 (7%)
**Preferred first-line treatment**		
	Onasemnogene abeparvovec	33 (81%)
	No preference	5 (12%)
	Combination onasemnogene abeparvovec and risdiplam	2 (5%)
	Risdiplam	1 (2%)
	Nusinersen	0 (0%)
**Most time-consuming step in initiating treatment**		
	Insurance approval	34 (83%)
	Genetic/laboratory testing	5 (12%)
	Time to referral	1 (2%)
	Time from insurance approval to treatment	1 (2%)

## Data Availability

The original contributions presented in the study are included in the article/Supplementary Materials, further inquiries can be directed to the corresponding author/s.

## Supplementary Materials

The following supporting information can be downloaded at: https://www.mdpi.com/article/10.3390/ijns10030058/s1.
