# Supplementary material for: Newborn Screening for Spinal Muscular Atrophy: Variations in Practice and Early Management of Infants with Spinal Muscular Atrophy in the United States
Source: Int J Neonatal Screen. 2024 Aug 16;10(3):58. doi: 10.3390/ijns10030058 (PMC11348092; doi:10.3390/ijns10030058)
Supplement: Supplementary file 1 [file IJNS-10-00058-s001.zip › IJNS-3068087-supplementary.pdf]

Supplement S1. Questionnaire administered to newborn state screening offices.

1. When did newborn state screening for spinal muscular atrophy (SMA) begin in your state? (exact date if possible)________

2. Total number of infants screened for SMA thus far (if only able to report total number of tests run please specify so) ______

(reporting period for this value _______)

3. How many patients have screened positive for SMA **with** confirmatory testing? ________ (reporting period for this value _______)

4. How many patients have screened positive for SMA **without** confirmatory testing? (if only able to report total number of tests please specify) _______

5. How many known false positive screening tests? ________

6. How many known false negative screening tests? ________

7. How many known SMA point mutations not detected on NBS? _______

8. What testing methodology is used for SMA screening in your state?
    a. Does the lab perform confirmatory testing?
    b. Does the lab run SMN2 copy number?
        i. If yes, is the lab able to differentiate between 4 copies and greater than 4 copes? what type of testing is used for this (qPCR, MLPA, NGS, ddPCR?)
    c. If no to either a or b, are there plans in place to adapt these testing methods?

9. If known, what is the Number of infants with each SMN2 copy number reported?

1 copy: ____
2 copies ____
3 copies _____
4 copies _____
5 copies _____
5+ copies_____

10. Once an infant screens positive for SMA, who is notified? (primary care provider, specialist, both?)

11. What information is provided to the primary care provider? Are we able to obtain a copy of the letter and factsheet sent to PCPs with information regarding the testing and treatment if one is sent?

12. What follow-up protocols are in place to verify results are received by primary care provider and/or specialist? Is there direct follow-up with PCP's office to ensure receipt?

13. What hospitals are these infants referred to for treatment ? Are we able to obtain the names and contact info of the specialists at these treatment centers? (***we would like to contact the specialists as part of our research project.***)

14. Does your office track information regarding treatment choices for infants screening positive for SMA? If possible, please share any treatment information about the identified individuals.

15. Is there any additional longitudinal follow-up performed by your office? If yes, what information is tracked?

16. Does your office participate in the NBSTRN tracking database?_______ Newsteps? _______ Others? _______

Follow up Questions

Does your office track the following information? If yes, please share these values if able.

1. How many infants have been referred to a specialist so far? ______
2. Average number of days from positive NBS screen to specialist referral ______
3. Average age of infant at time of specialist referral ______
4. Days from positive NBS screen to initiation of treatment ______
5. Average age of infant at time of treatment _______
6. Type of treatment received ______

Supplement S2: Survey of Providers

Are you the main care provider for newborns with SMA at your practice?

Yes (35, 85.4%), No- but I am able to answer questions regarding our practice's experience with newborns with SMA (6, 14.6%), No- please send this survey to the following provider (0, 0.0%).

Which of the following best represents your board certification/training background?

Adult Neurology (0, 0.0%), Adult Neurology with fellowship training in Neuromuscle or Electromyography (5, 12.2%), Child Neurology (7, 17.1%), Child Neurology with fellowship training in Neuromuscle or Electromyography (25, 61.0%), Genetics (0, 0.0%), General Pediatrics (0, 0.0%), Physical Medicine & Rehabilitation (1, 2.4%), Other (3, 7.3%: MDA care center director, General Pediatrics and Pulmonology, Child Neurology with Neuromuscular, EMG, and Genetics training)

Which of the following best describes the nature of your clinical practice?

Tertiary care center – academic (37, 90.2%), Tertiary care center – community hospital (2, 4.9%), Primary care or solo practice (0, 0.0%), Group practice (single specialty) (0, 0.0%), Group practice (multiple specialty) (2, 4.9%), Other (0, 0.0%)

In your clinical practice do you care for:

Primarily pediatric patients, with no or very rare adult patients (18, 43.9%), Mostly pediatric patients, with some adult patients (19, 46.3%), Roughly equal number of pediatric and adult patients (2, 4.9%), Mostly adult patients, with some pediatric patients (2, 4.9%), Primarily adult patients, with no or very rare pediatric patients (0, 0.0%)

Do you have admitting privileges to a pediatric hospital?

Yes (41, 100.0%), No (0, 0.0%)

Outside of residency/fellowship, do you regularly evaluate/provide care for newborns (age < 2 months) with neuromuscular disorders in your clinical practice?

Yes (40, 97.6%), No (1, 2.4%)

Outside of residency/fellowship, have you ever treated an infant (age < 1 year) with any of the following agents? (Check all that apply)

Spinraza (nusinersin) (40, 97.6%), Zolgensma (onasemnogene abeparvovec-xioi) (39, 95.1%), Evrysdi (risdiplam) (34, 82.9%), None of the above (1, 2.4%)

Is or was your primary site of clinical practice involved in any clinical trials related to SMA treatment in children?

Yes (19, 46.3%), No (22, 53.7%)

How many newborns (age < 2 months) have you evaluated for concern for SMA (including newborn screen positive referrals) in the past 24 months?

None (3, 7.3%), 1-5 (13, 31.7%), 6-10 (12, 29.3%), 11-15 (8, 19.5%), 16-20 (3, 7.3%), 21-25 (1, 2.4%), >25 (1, 2.4%)

What is the typical timeframe in which you will see a patient with a newborn state screen positive for SMA once a referral has been received?

Less than 72 hours (28, 68.3%), 4-7 days (10, 24.4%), Between 1-2 weeks (0, 0.0%), Between 2-3 weeks (0, 0.0%), Between 3-4 weeks (0, 0.0%), Greater than 4 weeks (0, 0.0%), I have not received any newborn screen SMA referrals (3, 7.3%)

Do you have a specific triage pathway in your practice when a referral for a newborn with possible SMA (newborn screen positive for SMA) is received?

Yes (39, 95.1%), No (2, 4.9%)

In newborns identified on newborn screening as having SMA, what is the average age of the infant at time of initiation of medical therapy?

Have not treated any infants (3, 7.3%), Less than 1 week of age (0, 0.0%), 1-2 weeks of age (6, 14.6%), 2-3 weeks of age (16, 39.0%), 3-4 weeks of age (11, 26.8%), 5-6 weeks of age (3, 7.3%), 6-7 weeks of age (0, 0.0%), 7-8 weeks of age (0, 0.0%), Greater than 8 weeks (2, 4.9%)

Is it your practice to perform repeat and/or confirmatory genetic testing for SMA in a NBS positive infant?

Yes (39, 95.1%), No (0, 0.0%), Our State Newborn Screen Lab already repeats/confirms the genetic testing (2, 4.9%)

Do you typically refer to other providers to arrange repeat/confirmatory testing?

Yes (2, 4.9%), No (39, 95.1%), I do not perform repeat/confirmatory testing for SMA (0, 0.0%)

When is repeat/confirmatory genetic testing for SMA typically initiated in your practice?

Prior to initial clinic visit (6, 14.6%), At time of initial clinic visit (34, 82.9%), Within 1 weeks of initial clinic visit (0, 0.0%), 1-2 weeks after initial clinic visit (0, 0.0%), More than 2 weeks after initial clinic visit (0, 0.0%), I do not perform repeat/confirmatory testing for SMA (1, 2.4%)

What do you perceive to be the most time-consuming step in the process of initiating treatment for infants with SMA?

Time to receipt of referral (1, 2.4%), Time from receipt of referral to patient appointment (0, 0.0%), Time needed for confirmatory laboratory/genetic testing (5, 12.2%), Insurance approval process (34, 82.9%), Time from insurance approval to treatment administration (1, 2.4%)

Which of the following represents your current first-line recommendations for therapy in newborn infants with SMA, assuming no contraindications to treatment?

Spinraza (nusinersin) (0, 0.0%), Zolgensma (onasemnogene abeparvovec-xioi) (33, 80.5%), Evrysdi (risdiplam) (1, 2.4%), Spinraza or Zolgensma or Evrysdi (all equivalent) (5, 12.2%), Dual therapy with Spinraza and Zolgensma (0, 0.0%), Dual therapy with Spinraza and Evrysdi (0, 0.0%), Dual therapy with Zolgensma and Evrysdi (2, 4.9%)

Please rank the following regarding decisions to start or defer treatment for SMA: Pre-symptomatic vs. symptomatic at time of evaluation, SMN2 copy number, Efficacy of treatment, Side effect profile/risks of treatment, Insurance authorization process, Cost

Please rank the following regarding your recommendation for selection of treatment agent:

Pre-symptomatic vs. symptomatic at time of evaluation, SMN2 copy number, Efficacy of treatment, Mechanism of action, Route/Frequency of administration, Side effect profile/risks of treatment, Insurance authorization process, Cost
